# Properties of Mosquito Repellent-Plasticized Poly(lactic acid) Strands

**DOI:** 10.3390/molecules26195890

**Published:** 2021-09-28

**Authors:** António B. Mapossa, Jorge López-Beceiro, Ana María Díaz-Díaz, Ramón Artiaga, Dennis S. Moyo, Thabang N. Mphateng, Walter W. Focke

**Affiliations:** 1 Institute of Applied Materials, Department of Chemical Engineering, University of Pretoria, Lynnwood Road, Private Bag X20, Hatfield, Pretoria 0028, South Africa; thabang.mphateng@gmail.com (T.N.M.); walter.focke@up.ac.za (W.W.F.); 2 UP Institute for Sustainable Malaria Control & MRC Collaborating Centre for Malaria Research, University of Pretoria, Private Bag X20, Hatfield, Pretoria 0028, South Africa; dsmoyorov@gmail.com; 3 Higher Polytechnical School, University of A Coruña (UDC), 15471 Ferrol, Spain; jorge.lopez.beceiro@udc.es (J.L.-B.); ana.ddiaz@udc.es (A.M.D.-D.); ramon.artiaga@udc.es (R.A.); 4 Institute of Applied Materials, Department of Chemistry, University of Pretoria, Lynnwood Road, Private Bag X20, Hatfield, Pretoria 0028, South Africa

**Keywords:** mosquito repellents, polymer strands, thermal analysis, rheology, desorption

## Abstract

Poly(lactic acid) (PLA) is an attractive candidate for replacing petrochemical polymers because it is fully biodegradable. This study investigated the potential of PLA as a sustainable and environmentally friendly alternative material that can be developed into commercially viable wearable mosquito repellent devices with desirable characteristics. PLA strands containing DEET and IR3535 were prepared by twin screw extrusion compounding and simultaneously functioned as plasticizers for the polymer. The plasticizing effect was investigated by thermal and rheological studies. DSC studies showed that the addition of DEET and IR3535 into PLA strands reduced the glass transition temperature consistent with predictions of the Fox equation, thus proving their efficiency as plasticizers. The rheology of molten samples of neat PLA and PLA/repellents blends, evaluated at 200 °C, was consistent with shear-thinning pseudoplastic behaviour. Raman studies revealed a nonlinear concentration gradient for DEET in the PLA strand, indicating non-Fickian Type II transport controlling the desorption process. Release data obtained at 50 °C showed initial rapid release followed by a slower, near constant rate at longer times. The release rate data were fitted to a novel modification of the Peppas-Sahlin desorption model.

## 1. Introduction

Malaria is a leading cause of illness and death in countries where the disease is endemic [1]. The disease is caused by the bite of mosquitoes carrying *Plasmodium* parasites [1]. Children younger than five years and pregnant women are most susceptible to malaria. In 2020, the World Health Organisation (WHO) reported around 229 million malaria cases with most occurring in sub-Saharan Africa [1]. There were approximately 409,000 deaths, including 272,000 children succumbing to the disease [1]. Strategies that target reduction in malaria transmission include long-lasting insecticidal nets (LLINs) and indoor residual spraying (IRS) [1]. These vector control interventions focus on minimizing malaria infections in indoor settings. However, they do not address the problem of malaria transmission in outdoor settings. A degree of personal protection is achieved by topical application of mosquito repellents [2]. Unfortunately, due to the high volatility of the repellents, this approach provides only short-term protection. This prompted the exploration and development of wearable products with longer-lasting mosquito repellence activity. For example, Sibanda et al. [3] developed polyolefin-based bicomponent fibres containing the repellent DEET. Socks containing these fibres provided effective protection against *An. arabiensis* for up to 20 weeks. Similarly, Mapossa et al. [4] investigated microporous polyolefin strands with either DEET or Icaridin trapped internally. Foot-in-cage bioassay results demonstrated effectiveness repellence of mosquitoes for up to twelve weeks. This offers the potential for the development of polymer-based anklets and bracelets providing prolonged protection of wearers against *An. arabiensis* bites.

Although these studies showed promising results, they employed nonbiodegradable petroleum-based polyolefins. The concern is that Africa is facing a growing waste management crisis. The volumes of waste generated in Africa, compared to developed regions, are relatively small. Nevertheless, the mismanagement of waste in Africa is already impacting human and environmental health [5]. Therefore, the present study and other associated investigations [6,7,8,9,10,11] opted to investigate poly(lactic acid) as an alternative polymer matrix for repellent incorporation.

PLA is a fully biodegradable, environmentally friendly polyester derived from renewable resources [9,12,13,14]. The lactic acid-based repeat unit contains carbon atom that is stereogenic. This means that polymer grades, constituted of different optically active forms, are available commercially. They include the homopolymers poly(l-lactic acid) and poly(d-lactic acid) in addition to random (d,l-lactic acid) copolymers (PDLLA) [11,15]. Generally, PLA features a high tensile strength and moisture and barrier properties similar to poly(ethylene terephthalate) (PET) [16]. Unfortunately, PLA tends to be very stiff and brittle at room temperature due to a high glass transition temperature (*T_g_*) of between 50 and 80 °C. This often limits its use in applications that require less brittle materials [17,18].

PLA strands intended for bracelet applications should preferably have a soft and rubbery feel. Such bracelets or anklets are expected to be aesthetically desirable and more comfortable to wear for the end-user. Hopefully, the repellents DEET and IR3535 that are to be trapped in the PLA matrix via extrusion compounding can also function as plasticizers for PLA. This could simultaneously improve the processability of the polymer and result in a more pliable material. Previous studies [17,19,20,21] found that the incorporation of plasticizers significantly reduced the glass transition temperature. This implies that improving the polymer flexibility should be possible. 

The ideal plasticizer for PLA is expected to be a polar, low-molecular-weight and nontoxic compound with a low volatility [21,22]. Mapossa et al. [23] examined the volatility of various mosquito repellents including ethyl anthranilate, DEET, citriodiol, dimethyl phthalate, decanoic acid and IR3535. It was found that DEET and IR3535 are among the least volatile repellents available. This prompted their evaluation as potential candidates for the “softening” of PLA in order to make it a more attractive matrix for long-lasting wearable mosquito-repellent devices. 

DEET is the key active ingredient in many commercial mosquito-repellent formulations. Despite assertions to the contrary in the popular press, DEET may be considered to be an environmentally friendly compound [19,24]. According to Weeks et al. [24], the bio-accumulation potential of DEET is low; it is neither a persistent bio-accumulating toxicant nor a persistent organic pollutant. This means that the probability for adverse effects to environmental species is low. Similarly, IR3535 is a particularly safe and effective repellent that has been available for more than twenty years [25]. Therefore, it is expected that neither of these repellents will negatively affect the biodegradability or compostability of PLA.

The incorporation of mosquito repellents into PLA was studied previously [6,7,8,9,10,11]. Di Lorenzo and Longo [19] prepared PLLA/DEET blends, containing up to 4.5 wt.% of the repellent, via melt extrusion. They noted a considerable reduction in the glass transition temperature even at this low DEET content and concluded that it is a very efficient plasticizer for PLLA. However, twin-screw compounding and profile extrusion of poly(lactic acid) strands containing higher DEET levels have not yet been investigated. This is addressed in the present study, with the focus on studying the plasticization effect of the mosquito repellents IR3535 and DEET at loading levels up to 25 wt.%. In this regard, the thermal and rheological properties of extrusion-compounded poly(lactic acid) strands were investigated. Finally, the release rate of the repellents from the extruded PLA strands was measured and modelled. Therefore, this communication reports on the potential of PLA to be a sustainable and environmentally friendly alternative material that could be developed into commercially viable, wearable mosquito-repellent devices with desirable characteristics. 

## 2. Modelling Diffusion-Controlled Repellent Release from Round Polymer Strands

Long strands with a circular cross-section, containing a fugitive repellent, can be modelled as infinite cylinders of diameter *d*. Several different cases were dealt with previously [4,26,27]. The first relates to repellents that dissolve and cause significant swelling of a polymer matrix that exists well above its glass transition temperature. That was the case for the EVA polymer studied by Sitoe et al. [26]. These strands shrank over time in step with the release of the repellents resulting in a significant decrease in both length and diameter. The second case relates to repellents incorporated in cellulose diacetate. In this case, the repellents also dissolved in the polymer, but very little shrinkage was observed. This was attributed to limited polymer chain segment mobility caused by the high glass transition temperature of the matrix which significantly exceeded the desorption temperature. The polyethylene strands studied by Mapossa et al. [28] did not suffer any shrinkage at all on release of the repellent. This was the case even though the matrix was far above its glass transition temperature. While the polar repellents were fully soluble in the polyethylene melt, the solubility was very low at room temperature. Phase separation, by spinodal decomposition, was induced by very fast cooling of the molten strands in an ice-cold water bath [4]. This triggered the formation of a finely structured microporous solid polymer scaffold which effectively immobilised the liquid repellent inside the strand. Additionally, the process led to the formation of a dense polymer skin layer covering the surface of the strands. Diffusion through this membrane was identified as the rate controlling step. The advantages of this process were that the strands did not shrink when the repellent was lost and that the release rate approached zero order kinetics [4]. 

The previous studies of the release of volatile actives from microporous polyethylene and solid cellulose diacetate strands concluded that the observed rates were consistent with the expectations for a diffusion-controlled process [4,27]. Presumably, this means that the release of repellents from the current PLA strands were also diffusion-limited. This is the case when the evaporation of the active from the surface into the environment is much faster than the rate at which it can be replenished by diffusion from inside the strand. Analytical solutions for this problem are available. However, they are all based on the assumption that the diameter of the strand remains constant during the process. Furthermore, the available analytic solutions are in the form of infinite series, and these converge very slowly. This makes them inconvenient to implement for correlating of experimental data. Therefore, Mphateng et al. [27] considered empirical and semi-theoretical release models [29,30,31,32,33]. Unfortunately, the models explored by Mphateng et al. [27] are not directly applicable to the current repellent-containing PLA strands. This is due to two complicating factors. Firstly, it was found that desorption of the repellents resulted in significant shrinkage of the PLA strands. Secondly, the initial repellent loading levels were sufficient to lower the glass transition temperature of the polymer to values below the test temperature of 50 °C. This means that the polymer, initially in a rubbery (or at least leathery) state, eventually reverted to a glassy state as repellent desorption proceeded to completion.

Both of the present liquid repellents can be considered to act as plasticizing solvents for the solid PLA polymer. The DSC results showed that the glass transition temperature decreased monotonically with increasing repellent content. Therefore, at ambient conditions, the repellents were fully dissolved in the PLA strands, i.e., the material was a polymer-rich, homogeneous single-phase system. Furthermore, the repellents appeared to be fully soluble up to the processing temperatures employed. This means that it was not possible to induce phase separation by spinodal decomposition for the repellent-PLA systems. 

As before, it is assumed that the repellent desorption is limited by the diffusional transport inside the polymer matrix. However, the process resulted in a rubber–glass transition, and this complicates the analysis. The concomitant viscoelastic relaxation effects have an impact on the rate of repellent migration, causing it to appear decidedly non-Fickian in nature. The physical mechanisms responsible for these phenomena are not yet fully elucidated but there is consensus that one important factor is a viscoelastic stress that can be as important to the transport process as the well-understood Fickian dynamics [34]. Furthermore, as desorption proceeds, a glassy skin may form at the exposed surface because the outer layer is expected to become depleted first. The skin may slow desorption of the repellents because the diffusion coefficient in the glassy region is much lower than in the rubbery region [35]. Over time, this layer grows thicker as more and more repellent is lost from inside. It is possible, and likely, that the transport of repellent across this vitrified layer represents the dominant factor limiting the rate at which the dissolved repellent migrates to the surface of the polymer. 

It is important to note that desorption of swelling solvents from swollen polymer samples presents several interesting features [36]. Initial rates of desorption are often significantly larger than absorption rates, while long-term desorption rates are extremely slow [36]. Thus, the characteristic feature of the second part of the problem of anomalous diffusion is the observation of higher rates of desorption when compared to those of sorption over the same concentration interval [36]. However, what is significant is the rapid decrease in the rate of desorption and the extremely long tail of the desorption curve [36].

Many different empirical models have been proposed to describe the overall desorption behaviour. Due to the complexities involved, none are fully predictive, and therefore, most require model fitting on experimental release data. Their utility lies in the fact that they facilitate the design of controlled release devices [37]. Release of an active ingredient via Fickian diffusion is known as Type I transport [38]. Type II transport is the anomalous migration effects observed when glassy polymers are involved [38]. The initial mass loss in thin films, due to Fickian diffusion, is proportional to the square root of time [38]. At a later stage, the rate becomes decidedly non-Fickian in nature with the time-exponent increasing to unity. According to Peppas and Sahlin [39], the overall behaviour is adequately described by an additive model with the time exponents taking on slightly different values for other geometries. For cylinders, they proposed the Peppas–Sahlin equation given here:

(1) $$\frac{M\left(t\right)}{M_{\infty }}={\left(\frac{t}{\tau_{1}}\right)}^{0.45}+{\left(\frac{t}{\tau_{2}}\right)}^{0.89}$$

The first term on the right-hand side *M*(*t*)/*M_∞_* describes the fraction of active ingredient released after time *t*. Note that *τ*_1_ and *τ*_2_ are characteristic rate-controlling time constants for the two desorption mechanisms.

## 3. Results and Discussion

### 3.1. Thermogravimetric Analysis

**Figure 1:**
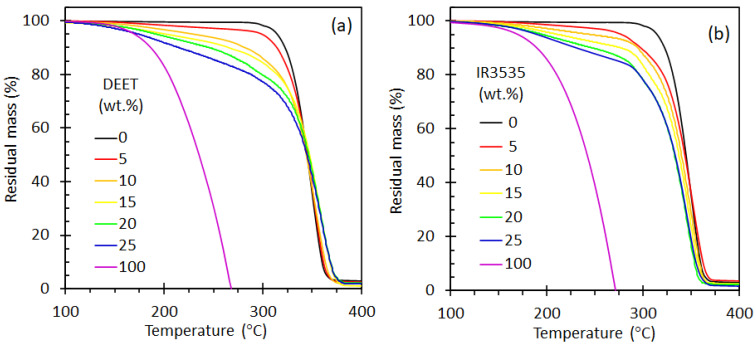
Thermogravimetric plots of: (**a**) neat DEET, neat PLA and PLLA/DEET blends; (**b**) neat IR3535, neat PLA and PLA/IR3535 blends. The legend indicates the nominal repellent contents of the PLA strands.

Figure 1 shows TGA traces for the neat mosquito repellents DEET and IR3535, the neat PLA as well as the compounds containing the active ingredients. The first mass loss event in all of the repellent-containing samples is attributed to the volatilization of the repellents. For the neat DEET, evaporation commenced at about 105 °C and was complete by 268 °C. By comparison, the evaporative mass loss of IR3535 started just above 108 °C and was complete by 270 °C. As expected, the mass loss curves indicate delayed volatilisation when the repellents were trapped inside the PLA matrix. The repellent mass loss was incomplete at the point where the PLA started to lose mass in earnest, i.e., above 294 °C. This means that it was not possible to accurately estimate the actual repellent content from the TGA data. Besides this, the incorporation of DEET and IR3535 did not materially delay the onset of mass loss of the PLA present.

### 3.2. Differential Scanning Calorimetry

**Figure 2:**
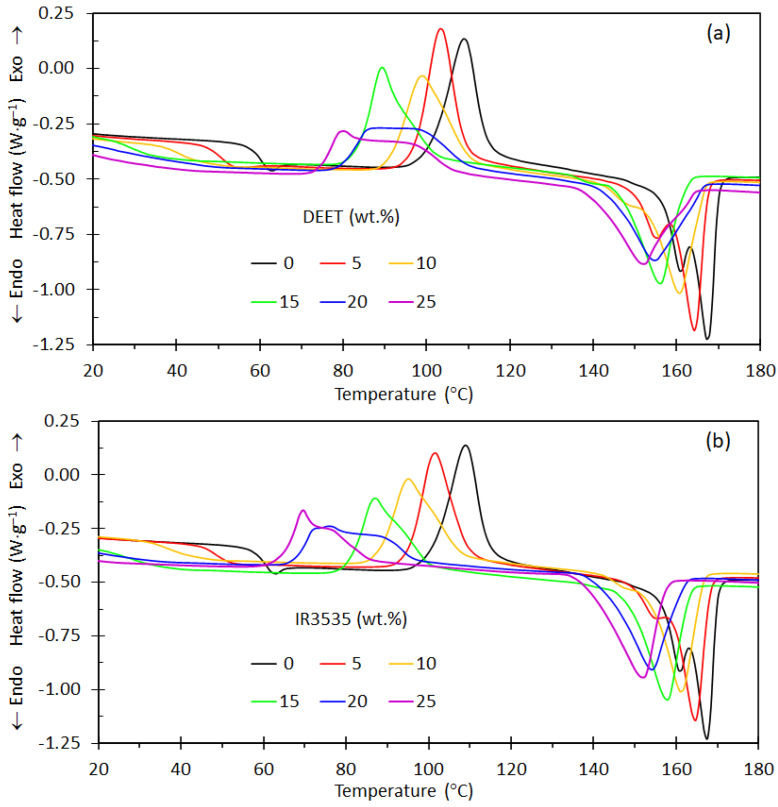
Differential scanning calorimetry traces obtained on the second heating scan for (**a**) neat PLA, PLA/DEET blends and (**b**) neat PLA, PLA/IR3535 blends.

Figure 2 shows the DSC results for the neat PLA and the PLA/DEET and PLA/IR3535 compounds recorded during a second heating scan. The step change in the heat flux seen at lower temperatures corresponds to the glass transition temperature. Shortly thereafter, exothermic recrystallization is observed, and at higher temperatures, the samples melt. The presence of the repellents changes the locations where these events occur. Table 1 reports the glass transition (*T_g_*) values together with the cold crystallization temperatures (*T*_cc_), the melting temperatures (*T*_m_), cold crystallization enthalpy (Δ*H*_cc_) and melting enthalpy (Δ*H*_m_). Two melting peaks are observed for the neat PLA as found previously by [40,41,42,43]. They correspond to the presence of the stable orthorhombic α crystalline form and the metastable α′-modification [44]. According to Righetti et al. [44], at the respective melting temperatures of 150 °C and 180 °C, the Δ*H*_m_ values of the α′- and α-forms are 107 and 143 J g^−1^. Conformational defects in the disordered α′-modification are responsible for its lower melting temperature and heat of melting.

Two distinct melting peaks are also observed for the PLA blend with 5 wt.% DEET or IR3535. The one on the lower-temperature side is less prominent. Just a vestige of a shoulder remained with 10 wt.% repellent present, and at even higher repellent concentrations, only a single melting peak is observed in Figure 2. The implication is that the presence of the repellents favours the formation of the *α* crystal form. Furthermore, the enthalpy of melting does not decrease in proportion to the polymer present in the blends. This indicates that higher levels of crystal formation happened when the repellents were present.

The observed decrease in the crystallization temperature with increasing repellent content can reportedly be modelled using the Flory equilibrium-melting-point depression equation [9]. The neat PLA re-crystallized on heating in the temperature range of 100–125 °C, with the maximum located at 109.0 °C. There was a downward shift in the temperature range of cold crystallization as the repellent content increased. This was probably caused by the very significant decrease in the glass transition temperatures with the concomitant increase in the mobility of PLLA chains [9]. According to Di Lorenzo and Longo [19], the enhanced mobility of PLLA chains upon addition of a repellent such as DEET affects the crystallization kinetics of PLLA, with a delayed nucleation upon melt crystallization but faster crystal growth rate as seen in the re-crystallization on heating. Ge et al. [45] observed similar effects of glycerol on the crystallization behaviour of PLA. 

**Figure 3:**
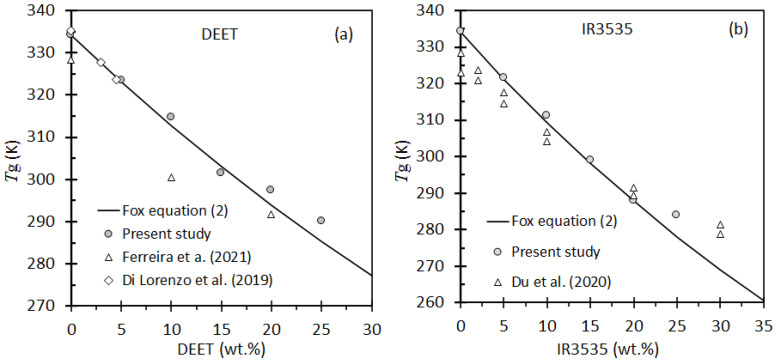
Experimental data for the glass transition temperatures for blends of PLA with (**a**) DEET and, (**b**) IR3535. The continuous line shows the trend, predicted by the Fox model, for the present data.

The glass transition (*T_g_*) values, listed in Table 1, were estimated from the positions of the inflection points in the DSC thermograms. The plasticizing effect of DEET and IR3535 on the PLA is indicated since the glass transition temperature shifted to significantly lower temperatures. The effect of repellent content on the *T_g_* is shown in Figure 3. The presence of a low-molecular-weight diluent resulted in an increase in the free volume of the system, thereby lowering *T_g_*. This effect is captured by the Fox equation [46] given here:

(2) $$\frac{1}{T_{g}}=\frac{w_{1}}{T_{g1}}+\frac{w_{2}}{T_{g2}}\;$$

where *w*_1_ and *w*_2_ are weight fractions to components 1 and 2, respectively, and their corresponding glass transition temperatures. Figure 3 indicates that the present results are broadly in agreement with the predictions of the Fox expression represented by Equation (2).

### 3.3. Raman Spectroscopy

**Figure 4:**
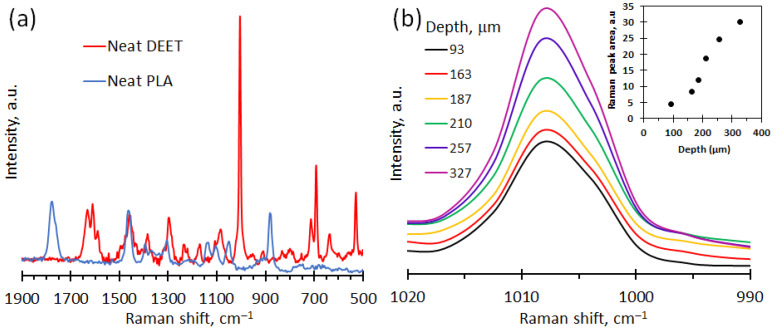
Raman spectra of: (**a**) Spectrum for neat DEET and neat PLA. (**b**) Confocal Raman spectra recorded at different positions inside a PLA strand initially containing 15 wt.% DEET. The distance from the surface indicated in units of μm. The insert shows integrated peak areas for the Raman band located between 1000 and 1020 cm^−1^ as a proxy for the concentration of DEET inside the PLA strand.

Figure 4a shows an overlay of the Raman spectra of neat PLA and neat DEET. The strong band located at 1004 cm^−1^ is unique to DEET because it is not present in the spectrum of PLA. Figure 4b shows the variation of Raman intensity as a function of depth (µm) into the PLA strand. On incorporation into the PLA polymer at a concentration of 15 wt.%, the peak unique to DEET shifted to 1008 cm^−1^. The shift suggests that the repellent was dissolved in the polymer. The inset in Figure 4b shows the variation of the area under this 1008 cm^−1^ Raman band. It represents a proxy for the concentration of DEET in the polymer [3]. The observed DEET concentration, within the PLA strand, features an S-shaped concentration dependence on the penetration depth. The highly nonlinear variation in the radial direction is clearly non-Fickian in nature. Rather, it suggests that Type II transport is controlling the desorption process. As Edwards [47] indicated, the desorption of a migrating compound from a polymer matrix which reverts to a glassy state during the process is complex. It involves a moving boundary at the glass–rubber transition which separates the polymer into two regions. In the inner region, the concentration of the migrating species is high, and the material is rubbery in nature. Near the surface, the concentration is low, and the material assumes a glassy state. The phase morphology in each of these regions affects the diffusional transport in different ways. 

### 3.4. Rheological Properties

**Figure 5:**
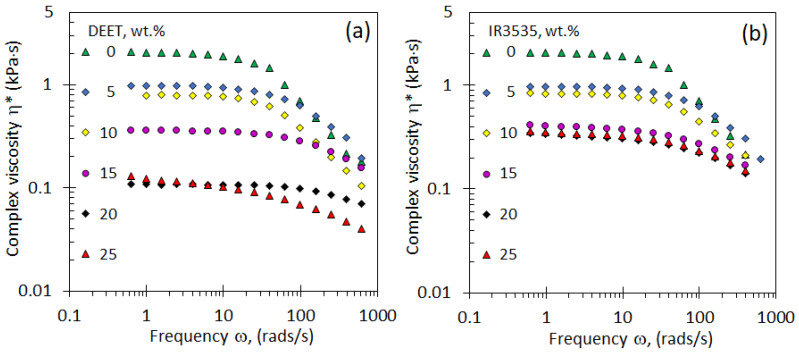
Complex viscosity vs. versus angular frequency curves measured at 200 °C. The effect of repellent concentration is shown in (**a**) for PLA/DEET blends, and in (**b**) PLA/IR3535 blends.

**Figure 6:**
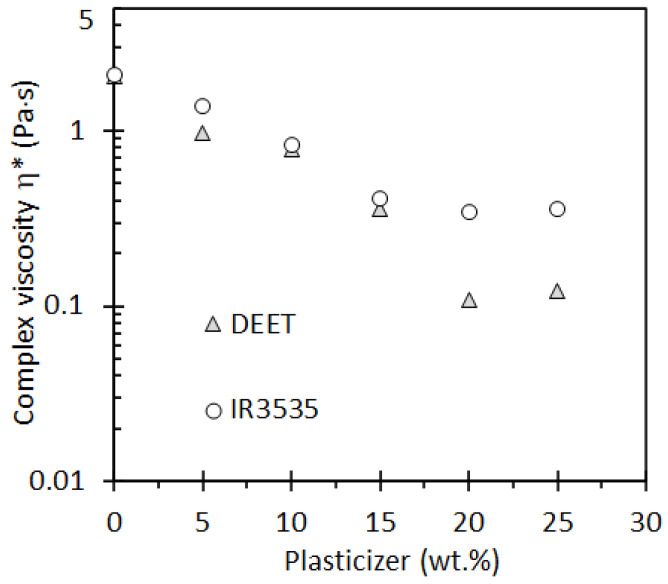
Variation of the complex viscosity with the concentration of the repellents DEET and IR3535. The plotted plateau values reflect measurements obtained at 200 °C.

The effect of the presence of the repellents on the melt viscosity of the PLA is important for the preparation of strands by extrusion processes. Figure 5 shows the effect of repellent content and angular frequency on the complex viscosity of the blends. It shows data obtained at a fixed temperature of 200 °C, a likely processing temperature. Plateau values are observed at low frequencies consistent with Newtonian behaviour. In this region, addition of a repellent causes a large drop in the viscosity as seen in Figure 6. For DEET, the complex viscosity of the melt decreases by more than one order of magnitude as the repellent content is increased to 25 wt.%. Adding IR3535 also causes a significant reduction in the melt viscosity, but the drop is less pronounced. The neat polymer and the blends all show shear-thinning non-Newtonian behaviour at higher angular frequencies. The slope is most negative for the neat PLA, and it becomes less steep as more repellent is present. This progressive convergence means that the differences between the viscosities of the blends and that of the neat polymer are less pronounced at high angular frequencies. 

### 3.5. Repellents Release from PLA Strands

The average in initial and final diameters of the strands after five months of ageing in a convection oven at 50 °C are reported in Table 2. The data indicates that the loss of the repellents was associated with significant shrinkage. Therefore, the assumption of a constant strand diameter is not valid.

**Figure 7:**
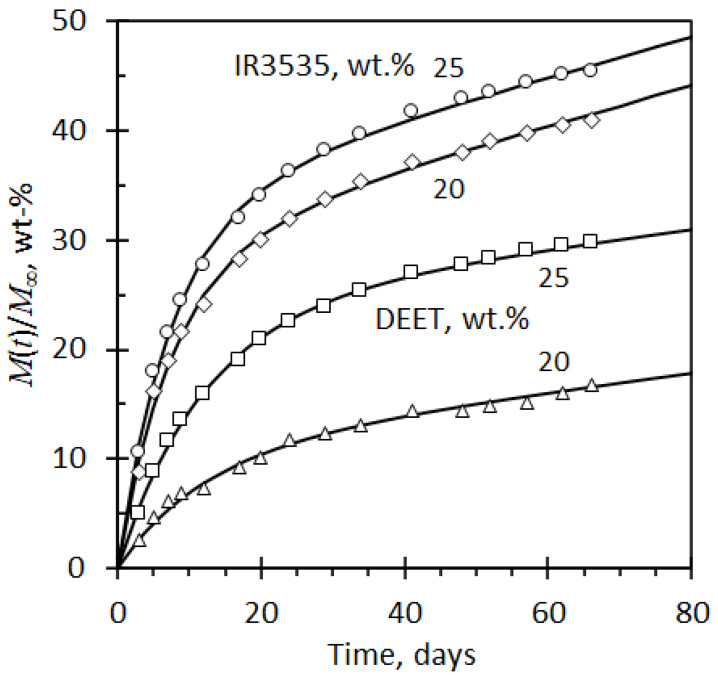
Repellent release data for DEET and IR3535 trapped inside cylindrical PLA strands. The initial concentrations of the repellents are indicated. The data reflects measured mass loss data recorded on ageing blends in convention oven controlled at 50 °C over a period of 66 days. The solid lines represent prediction data obtained by Equation (3) based on the regression constants presented in Table 3.

Figure 7 shows the release curves for DEET and IR3535 from the PLA strands as a function of time. IR3535 was lost more rapidly than DEET, and, at any given time, the fractional release was higher for strands containing a higher repellent loading. Regression attempts showed that the present data did not fit the Peppas–Sahlin model, i.e., Equation (1). It was found that the initial release rate was significantly faster that indicated by a square root time dependence. However, very good fits were obtained using the following empirical modification demonstrated by Equation (3):

(3) $$\frac{M\left(t\right)}{M_{\infty }}=X_{1}\left[1-\exp\left(-\frac{t}{\tau_{1}}\right)\right]+{\left(\frac{t}{\tau_{2}}\right)}^{0.89}$$

In this equation, the second term of the Peppas–Sahlin model was retained, whereas the first term was replaced by an exponential relaxation expression, with *X*_1_ indicating the fractional contribution this term makes to the overall release via desorption. Nonlinear least-squares regression revealed that the characteristic time constants were not affected by the repellent loading level, but they did differ according to the nature of the repellent present. Therefore, the data for each repellent were regressed together with only the *X*_1_ parameter allowed to differ for the two separate repellent loadings used. Table 3 lists the values obtained for the adjustable parameters of Equation (3), and the predicted regression behaviour is indicated by the solid lines shown in Figure 7. The *X*_1_ values are larger for higher loadings of the repellents. This indicates that the initial release mechanism increases in importance with increase in repellent content.

## 4. Materials and Methods

### 4.1. Materials

The mosquito repellent liquids *N,N*-diethyl-3-methylbenzamide (DEET) (purity 97%) [CAS-No. 134-62-3] and ethyl butylacetylaminopropionate (trade name IR3535) (purity ≥ 99%) [CAS-No. 52304-36-6] were supplied by Sigma-Aldrich and Merck respectively. DEET exhibits a glass transition temperature (*T_g_*) of around 198 K [48] while IR3535 has a glass transition temperature (*T_g_*) of around 185 K and change of the specific heat capacity (Δ*C*p, IR3535 (*T_g_*)) of 0.64 J/(g K) [8]. All compounds were used as received, i.e., without further purification. A commercial semi-crystalline fibre grade poly(lactic acid) was used in the extrusion-compounding experiments. The Institute of Polymer Research in Dresden, Germany assisted with the characterization of this polymer sample. The molecular mass was determined with size exclusion chromatography (SEC) using chloroform as the mobile phase. The weight-average molar mass was 130 kDa and the number-average molar mass was (Mn) 44 kDa corresponding to a polydispersity of 2.95. The D-unit content was determined as 2.3% by polarimetry. The optical rotation of a 1.00 g dL^−1^ solution of the polymer was measured at a wavelength of 589 nm. 

### 4.2. Extrusion-Compounding

Before processing, the pulverized PLA was dried for 48 h at 60 °C in a convection oven. This was done to avoid polymer hydrolysis during the extrusion-compounding operations. The required amounts of the PLA powder and a repellent were weighed into a plastic pail. The ingredients were manually ladled into a semi-dry homogeneous blend that could be fed into the compounding extruder. The extrusion-compounding was performed on a TX28P 28 mm corotating twin-screw laboratory extruder manufactured by CFAM Technologies, Potchefstroom, South Africa. The screw diameter was 28 mm, and the L/D ratio was 18. The screw design comprised intermeshing kneader blocks that also impart a forward transport action. The temperature profile, from hopper to die, was set as follows: 140/160/175/175 °C. The extruder screw speed was varied from 80 to 100 rpm. The exiting PLA strands were directly extruded into an ice-cold water bath. The diameters of the extruded strands were controlled at ca. 4 mm.

### 4.3. Thermogravimetric Analysis (TGA)

The thermal behaviour of the neat PLA and its combinations with the repellents was investigated with a TA Instruments 2960 SDT simultaneous TGA-DSC instrument (TA Instruments, New Castle, DE, USA) Samples, weighing between 10 to 11 mg, were heated from ambient temperature to 600 °C at a rate of 10 K⋅min^−1^. The purge gas was nitrogen flowing at 100 mL⋅min^−1^. The first weight-loss step of the polymer strand was associated with the loss of the repellent due to volatilization. This event partially overlapped with a subsequent polymer degradation step.

### 4.4. Differential Scanning Calorimetry (DSC)

DSC scans were performed on a TA Instruments Q2000 MDSC. The samples were placed into Tzero aluminium crucibles that were crimped closed. The temperature calibration of the DSC instrument (TA Instruments, New Castle, DE, USA) was performed according to the manufacturer recommendations. The experiments consisted of linear heating ramped at 10 K⋅min^−1^ up to 200 °C followed by a 2 min isothermal step and then a cooling ramp at 10 K⋅min^−1^ down to a temperature well below the glass transition. A second 10 K⋅min^−1^ heating ramp was applied up to 200 °C. Only the melting events recorded in the second heating cycle are reported. The glass transition temperatures of the PLA-repellents compounds were estimated from the position of the inflection point observed in the DSC traces.

### 4.5. High-Resolution Confocal Raman Imaging

First, Raman spectra of the pure materials were recorded with a WITec confocal Raman microscope (WITec Alpha 300R, Ulm, Germany). The 532 nm excitation laser was operated at power of 10 mW and the acquisition time was 2 × 30 s. Then the high-resolution confocal Raman spectroscope was employed to probe the concentration profile of DEET within an extruded PLA strand containing 15 wt.% of the repellent after four months from date of preparation. Single spectra were recorded at different depths and the intensity of a characteristic peak of DEET was used as proxy for its concentration at different scan depths. 

### 4.6. Rheological Tests

Rheological measurements of the blends were conducted at a temperature of 200 °C on a Discovery HR-2 rheometer. Strain and frequency sweeps on the molten samples were carried out using the 25 mm φ parallel plate geometry. For each sample, strain sweeps were performed at 10 Hz in order to establish the linear range. The strain applied during frequency sweeps was chosen to be inside the linear range. The angular frequency range used during measurement was 0.1–1000 rad⋅s^−1^. The storage modulus (G′) and loss modulus (G″) were measured at various strain amplitudes at a frequency of 10 Hz.

### 4.7. Mosquito Repellent Release Studies

Oven ageing tests were conducted to track the time-dependent mass loss of the repellents from the PLA strands. In these experiments, the strands were suspended vertically in a convection oven set at 50 °C. The mass of the strands was measured twice a week, reaching a total of sixteen measurements after an ageing period of sixty-six days. At this point, COVID lockdown regulations began, and, unfortunately, the experiments had to be terminated prematurely. All laboratory instruments and ovens had to be shut down for safety reasons for several weeks. However, thereafter, the strands were again aged at the same conditions for a total of five months. The initial and final diameters of the strands were measured with Mitutoyo Digital Vernier calliper.

## 5. Conclusions

Poly(lactic acid) strands containing up to 25 wt.% of the mosquito repellents DEET or IR3535 were successfully prepared by twin-screw extrusion-compounding process. The reduction of the polymer glass transition by the presence of the repellents accorded with the predictions of the Fox equation. This indicates that the process delivered homogeneous, polymer-rich single-phase materials. The melts showed pseudoplastic rheological behaviour at 200 °C with the fluidity improving with repellent content. The desorption rates measured at 50 °C showed fast initial release behaviour that slowed down to a near steady rate at longer times. This accords with Type II transport controlling the desorption process. This notion is supported by the highly nonlinear concentration gradient observed by confocal Raman studies for DEET inside a PLA strand. These results promise the possibility of developing long-life wearable mosquito-repellent devices such as bracelets and anklets from commercially available and biodegradable PLA, which can serve as an environmentally friendly alternative to existing petroleum-based devices such as polyethylene. Such anklets and bracelets may have utility for outdoor protection against infectious mosquito bites in malaria-endemic regions.

## Figures and Tables

**Table 1:** DSC Parameters obtained for neat PLA, PLA/DEET and PLA/IR3535 blends.

Repellent	(wt.%)	*T_g_* (°C)	*T*_oc_ (°C)	*T*_pc_ (°C)	*T*_om(1)_ (°C)	*T*_pm(1)_ (°C)	*T*_om(2)_ (°C)	*T*_pm(2)_ (°C)	Δ*H*_cc_ (J⋅g^−1^)	Δ*H*_m_ (J⋅g^−1^)
None	0	61.0	100.9	109.0	156.4	160.8	162.2	167.5	31.2	34.9
DEET	5	50.2	97.0	103.3	150.2	155.1	158.3	164.3	29.5	33.7
	10	41.5	91.3	98.8	142.6	150.8	151.7	160.6	28.9	31.8
	15	28.3	83.7	89.2	134.6	140.9	145.8	155.9	21.8	30.8
	20	24.3	79.8	88.7	-	-	143.3	154.5	26.8	30.0
	25	17.0	74.5	79.9	-	-	139.8	152.0	25.3	29.2
IR3535	5	48.5	95.2	101.6	148.9	155.9	158.4	164.6	26.9	33.3
	10	38.0	88.1	95.0	142.7	148.0	153.2	161.2	27.3	31.9
	15	25.9	80.9	86.8	136.9	142.1	148.4	157.6	22.9	29.9
	20	14.9	67.9	76.0	-	-	142.4	154.1	20.9	30.1
	25	10.7	64.7	69.5	-	-	138.7	151.5	17.8	30.7

*T_g_* = Glass transition temperature; *T*_cc_ = Cold crystallization temperature; *T*_m_ = Melting point; *T*_oc_ = onset crystallization temperature; *T*_pc_ = Peak crystallization temperature; *T*_om(1)_ and *T*_om(2)_ = Double onset melting temperatures; *T*_pm(1)_ and *T*_pm(2)_ = Double melting peaks temperature.

**Table 2:** Shrinkage of PLA-repellent strands expressed in wt.% evaluated at 50 °C.

Strand Type	Initial Diameter (mm)	Final Diameter (mm)	Difference (%)
Neat PLA	3.3 ± 0.1	3.2 ± 0.1	3.0
IR3535 (20 wt.%)	4.3 ± 0.5	3.9 ± 0.4	9.3
IR3535 (25 wt.%)	4.6 ± 0.3	4.0 ± 0.4	13.0
DEET (20 wt.%)	3.1 ± 0.1	2.7 ± 0.2	12.9
DEET (25 wt.%)	3.5 ± 0.1	2.9 ± 0.1	17.1

**Table 3:** Parameter for the desorption model of Equation (3) obtained by nonlinear least squares regression for release data obtained for PLA-repellent aged at 50 °C.

Strand Type	*X*_1_ (-)	*τ*_1_ (Days)	*τ*_2_ (Days)	*R*
IR3535 (20 wt.%)	27.6	7.66	607	0.9982
IR3535 (25 wt.%)	32.1	7.66	607	0.9986
DEET (20 wt.%)	9.96	12.0	1397	0.9962
DEET (25 wt.%)	23.1	12.0	1397	0.9993

*R* = Correlation coefficient.

## Data Availability

Data are contained within the article.

## Sample Availability

The data presented in this study are available on request from the corresponding author.
